# Phylogenetic Correlation and Symbiotic Network Explain the Interdependence Between Plants and Arbuscular Mycorrhizal Fungi in a Tibetan Alpine Meadow

**DOI:** 10.3389/fpls.2021.804861

**Published:** 2021-12-17

**Authors:** Qiang Dong, Xin Guo, Keyu Chen, Shijie Ren, Muhammad Atif Muneer, Jing Zhang, Yaoming Li, Baoming Ji

**Affiliations:** ^1^School of Grassland Science, Beijing Forestry University, Beijing, China; ^2^College of Resources and Environment, International Magnesium Institute, Fujian Agriculture and Forestry University, Fuzhou, China

**Keywords:** arbuscular mycorrhizal fungi, plant species, host selectivity, symbiotic network, phylogenetic distance, community dissimilarity, Tibetan alpine meadow

## Abstract

Plants and arbuscular mycorrhizal fungi (AMF) can form complex symbiotic networks based on functional trait selection, contributing to the maintenance of ecosystem biodiversity and stability. However, the selectivity of host plants on AMF and the characteristics of plant-AMF networks remain unclear in Tibetan alpine meadows. In this study, we studied the AMF communities in 69 root samples from 23 plant species in a Tibetan alpine meadow using Illumina-MiSeq sequencing of the 18S rRNA gene. The results showed a significant positive correlation between the phylogenetic distances of plant species and the taxonomic dissimilarity of their AMF community. The plant-AMF network was characterized by high connectance, high nestedness, anti-modularity, and anti-specialization, and the phylogenetic signal from plants was stronger than that from AMF. The high connected and nested plant-AMF network potentially promoted the interdependence and stability of the plant-AMF symbioses in Tibetan alpine meadows. This study emphasizes that plant phylogeny and plant-AMF networks play an important role in the coevolution of host plants and their mycorrhizal partners and enhance our understanding of the interactions between aboveground and belowground communities.

## Introduction

Arbuscular mycorrhizal fungi (AMF) are an ancient root symbiotic group, whose origin is consistent with the first appearance of terrestrial plants, and generally considered to be the result of coevolution of fungi and plants (Bonfante and Genre, 2008; Walder and van der Heijden, 2015; Genre et al., 2020). As a key component of the underground biological community, AMF can form a symbiotic relationship with most terrestrial plant species and can increase the absorption of phosphorus and nitrogen for plants (Wang and Qiu, 2006; Smith and Read, 2008; Zhang et al., 2021). At the same time, plants provide photosynthetic carbon products to AMF (Bever et al., 2009; Kiers et al., 2011; Wipf et al., 2019). The combination of AMF and plants improves the adaptability of plants and AMF symbionts (Blackwell, 2000; Chen et al., 2018; Tedersoo et al., 2020). Furthermore, the plant-AMF symbioses are not formed stochastically but are dependent on their functional traits (Bever et al., 2009; Kiers et al., 2011). Both plant hosts and AMF have been shown to preferentially allocate resources to higher-quality partners (Ji and Bever, 2016), inducing reciprocal shifts in each assemblage, ultimately affecting the partner selection in the mycorrhizal symbiosis (Werner and Kiers, 2015). Due to the simultaneous processes of coevolution, niche differentiation, and niche conservatism, the closely related plants tend to interact with similar AMF species (i.e., phylogenetic signal in the interaction) (Gomez et al., 2010; Sargent et al., 2011). However, negative correlations between the phylogenetic distance of plants and AMF community distance were also reported in three locations of the northern Great Plains of the United States (Reinhart and Anacker, 2014). In addition, the specificity of the interactions between the plant and AMF changed with community succession (Montesinos-Navarro et al., 2012a). These contrasting findings indicate the complexity of plant and AMF interactions.

Many studies have shown that phylogenetic relatedness affects the mycorrhizal symbiotic network structure, which could answer the question of how coevolution explained species assemblages (Barberan et al., 2012; Chagnon et al., 2012; Montesinos-Navarro et al., 2012b; Chen et al., 2017; Goberna et al., 2019). The network is increasingly used to study co-occurrence patterns in plant and AMF symbioses, providing insights for biological interactions (Barberan et al., 2012; Chagnon et al., 2012; Chen et al., 2017; Gao et al., 2019). The AMF communities of 166 root samples from 17 woody plants in subtropical forests of China showed that the woody plant-AMF network was highly interconnected and nested but in anti-modular and anti-specialized manners (Chen et al., 2017). Most of the studies on the plant-fungus symbiotic network focused on temperate and tropical ecosystems, and the research objects were mainly woody plant-AMF symbiotic interactions. However, herbaceous plant-AMF network characteristics in the alpine ecosystems remain poorly understood.

As the Third Pole on the earth, the Tibetan Plateau is a unique geographic unit with great biodiversity and critical ecosystem functions and is experiencing striking climate warming (Bai-ping et al., 2002; Duan et al., 2006; Qiu, 2008). Alpine meadow is a typical vegetation type, accounting for approximately 40% of the total grassland area on the Tibetan Plateau (Miehe et al., 2008; Wang et al., 2016). The importance of AMF in plant succession on the Tibetan plateau has been well documented (Liu et al., 2011; Gai et al., 2012; Li et al., 2015). However, most of the studies focused on the effects of altitude gradients (Liu et al., 2011; Gai et al., 2012; Li et al., 2014), precipitation (Zhang et al., 2016), and vegetation types (Gai et al., 2009; Gao and Guo, 2010; Xu et al., 2017) on AMF community composition. The selectivity of the interactions between the plant and AMF and their network properties in Tibetan alpine meadows remains unclear.

In this study, to explore the selectivity in the plant-AMF interactions and clarify the maintenance mechanism of symbiosis, 23 species of common herbs in a Tibetan alpine meadow were selected. Illumina-MiSeq sequencing was used to characterize AMF communities living in each plant root to address the following questions: (1) How is the selectivity in the plant-AMF interactions in Tibetan alpine meadow? and (2) What are the characteristics of the plant-AMF network in Tibetan alpine meadows?

## Materials and Methods

### Site and Sampling

The study site is located at the Naqu Alpine Grassland Ecosystem National Field Scientific Observation and Research Station (Kaima Station) of Tibetan Plateau Research of Chinese Academy of Sciences (31°16′N, 92°06′E, 4453 m a.s.l). This region is a typical alpine meadow ecosystem, where the mean annual temperature is −2.1°C, the mean annual rainfall is 406 mm, and most of the precipitation was concentrated from June to September. Summers are short and cool, and winters are long and severely cold (Cui et al., 2017). The plant community is dominated by *Kobresia Pygmaea* and *Kobresia Humilis*, and the *Carex moorcroftii*, *Stipa purpurea*, *Poa tibetica*, *Potentilla bifurca*, and *Heteropappus semiprostratus* (Hs) are companion species.

**FIGURE 1:**
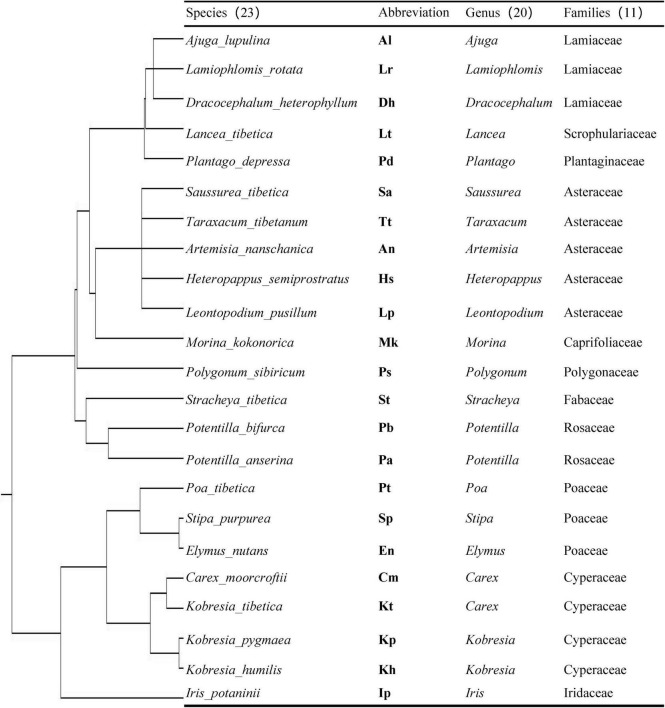
Phylogenetic tree of 23 plants species constructed using Phylomatic. The detailed information of the 23 plant species is shown including their abbreviation, genus, and families.

In mid-August 2018, 23 representative plant species (Figure 1) were selected based on a vegetation survey in the alpine meadow of Naqu county. Notably, 23 sampling sites were randomly chosen for plant sampling around the Kaima Station, which was at least 50 m away from each other to avoid interactions among plant species. Then, three 0.5 × 0.5 m^2^ quadrats were randomly selected in each sampling site, and three plant individuals in each quadrat were collected and pooled as one composite sample. Finally, 207 plant individuals of 69 samples (i.e., 23 plant species × 3 replicates) belonging to 11 families and 20 genera (Figure 1, according to the genetic relationship of species) were collected in this study. Plant roots were carefully separated from aboveground parts and soil, packaged in plastic bags, refrigerated and transported to the laboratory, and stored at −2°C for colonization determination and DNA extraction.

### Plant Phylogenetic Tree

Based on Zanne phylogeny as the backbone (Zanne et al., 2014), the phylogenetic tree of plant species was constructed using Phylomatic (V.3)^1^. The phylogenetic distance of plant species was calculated using R software 3.1.1 with the package “picante” (V.1.8.2) (Kembel et al., 2010).

### Arbuscular Mycorrhizal Fungi Colonization

The plant roots were washed carefully and cut into 1 cm length. Approximately, 100 root segments of each sample were cleared in 10% KOH at 90°C for 90 min, then acidified in 2% HCl at room temperature for 10 min, and stained with 0.05% Trypan blue at 90°C for 10 min. Finally, the dyed root segments were randomly selected and measured for the mycorrhizal colonization by the magnified intersections method at 200 × microscopes (McGonigle et al., 1990).

### DNA Extraction and Polymerase Chain Reaction

Total DNA was extracted from a 0.05 g (freeze-dried) subsample of fine roots using a PowerPlant DNA Isolation Kit (MoBio Laboratories, United States) following the instructions of manufacturer. A NanoDrop 8000 Ultramicro UV spectrophotometer (NanoDrop Technologies, Wilmington, DE, United States) was used to determine the quantity and purity of DNA samples. A two-step polymerase chain reaction (PCR) was performed to amplify the 18S ribosomal RNA (rRNA) gene. The primers used in the first PCR were NS31 (TTGGAGGGCAAGTCTGGTGCC) and AML2 (GAACCCAAACACTTTGGTTTCC) and the second PCR amplification with the primers AMV4.5NF (AAGCTCGTAGTTGAATTTCG) and AMDGR (CCCAA CTATCCCTATTAATCAT) (Lumini et al., 2010; Van Geel et al., 2014). The PCR products were purified with an agarose gel DNA purification kit (AP-GX-250G; Axygen, United States) and quantified using a NanoDrop 8000. The purified products were pooled in equimolar amounts and then sequenced on the Illumina-MiSeq platform (Shanghai Magigene Inc., Shanghai, China).

### Bioinformatics Analysis

We used the Quantitative Insights Into Microbial Ecology (QIIME) and the UPARSE pipeline to treat raw sequences as described previously (Caporaso et al., 2010; Edgar, 2013). Sequence quality control was conducted with QIIME, and the obtained high-quality sequences were imported into a Usearch (vision 11). Then, the Usearch was used for dereplication and merging of paired-end reads. The singletons were removed, and the chimeras were detected and removed using the *de novo* approach. The operational taxonomic units (OTUs) were clustered at 97% similarity. The representative sequences of OTUs were uploaded to the National Center for Biotechnology Information (NCBI) and MarrjAM^2^ database for taxonomy identification. Non-AMF reads were removed from the OTU table. Then, the OTU table was rarefied to a sequencing depth of 5,000 reads per sample, resulting in 345,000 AMF reads for all samples (i.e., 69 samples × 5,000 reads = 345,000 AMF reads). The rarefaction curves of each sample tended to be flat, indicating adequate sequencing depth (Supplementary Figure 1). Alpha diversity metrics (richness and Shannon-Wiener) were calculated using the rarefied OTU table. The phylogenetic tree of AMF OTUs was constructed according to Neighbor-Joining using MEGA 7.0 software (Supplementary Figure 2). The sequences obtained in this study have been submitted to the GenBank database (PRJNA 748864).

### Statistical Analysis

Analysis of variance (ANOVA) test and Tukey’s honestly (HSD) significant difference test (*P* < 0.05) were used for the pairwise comparisons of the diversity of AMF in different plant species. We tested for host-plant species effects on AMF community dissimilarity using Permutational ANOVA (PERMANOVA) using the “adonis” function in the R package “vegan” (V.2.5.7) with 999 permutations. Mantel tests were used to examine the correlation between AMF community dissimilarity matrix (Bray-Curtis and Unifrac distance) and plant phylogenetic distance matrix, and the relationship between pairwise AMF community dissimilarity distances and plant phylogenetic distances was tested by linear regressions. To better explain the plant-AMF symbiotic network, the plant-AMF visualization matrix at the species level was established. For a given paired plant-OTU sample, the association frequency (i.e., 0, 1, 2, or 3) was defined as the number of plant root samples with AMF OTUs observed (Toju et al., 2016), which reflected the selectivity of plant-AMF association. The association frequency matrix was represented by the heat map using TBtools software (v.0.6655) (Chen et al., 2020).

The species-level matrix was used to calculate the network indexes, such as connectance, nestedness, modularity, specialization, and checkerboard score. The ecological meaning of these indexes can be found in the Supplementary Material.

With network analysis and null model comparison, we could infer the non-randomness constructional laws of biological communities (Ulrich and Gotelli, 2007). The result of the network structure index was stable using the random test. First, random values of 1,000 randomly arranged samples of the null model matrix were generated, and then the *t*-test (*P* < 0.05) was used to test the significant difference between the observed values and the random values based on the null model. Based on the association frequency matrix and random matrix, weighted connectance, *H_2_′*, weighted nestedness metric based on the overlap and decreasing fill (WNODF), and checkerboard score index were calculated using “bipartite” package “networklevel” command in R (Dormann et al., 2013). For the modular analysis, the association frequency matrix and random matrix were the outputs from R, and then the data were analyzed using the MODULAR program. The estimated generalized least squares method (EGLS) was used to assess the phylogenetic signals of plant-AMF. The analysis fitted the phylogenetic variance-covariance matrix to the plant-AMF association frequency matrix (Ives and Godfray, 2006; Montesinos-Navarro et al., 2012b). Using the EGLS method, we calculated the independent phylogenetic signals of the plant (*d*plant) and AMF (*d*AMF) on association frequency matrix, assessed the overall intensity of the two phylogenetic signals combined with the mean square error (MSE) value, and regarded the model of minimizing MSE as the best fit. The MSE full model (MSEd), the MSE derived under the assumption of no phylogenetic signal (i.e., a star phylogeny, MSEstar), and maximum phylogenetic signal (i.e., Brownian motion evolution, MSEb) were calculated according to the description of Chen et al. (2017).

## Results

### Arbuscular Mycorrhizal Fungi Colonization, Diversity, and Community Composition of Different Plant Species

The sequences annotated as AMF were grouped into 121 OTUs, covering 3 orders, 6 families, and 9 genera (Supplementary Figure 3 and Supplementary Table 1). *Glomus* was the dominant genus (181013 sequences corresponding to 52.5% of the total sequences and 84 OTUs corresponding to 69.4% of the total OTUs), followed by *Claroideoglomus* and *Rhizophagus* (Supplementary Table 1).

**FIGURE 2:**
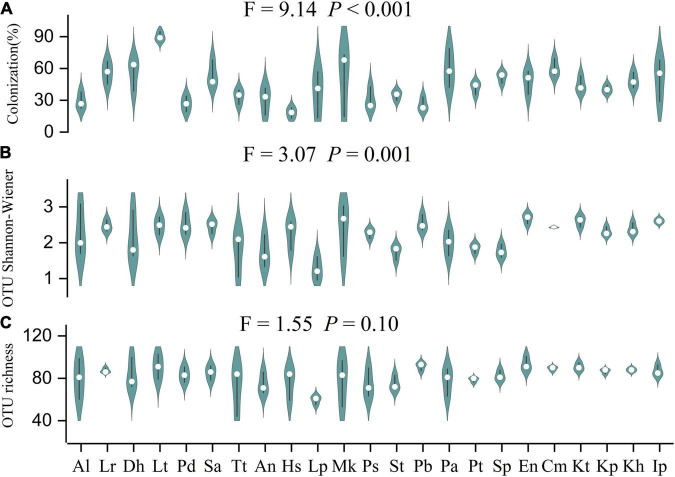
Differences in Arbuscular mycorrhizal fungi (AMF) colonization **(A)**; OTU Shannon-Wiener diversity index **(B)**; and OTU richness **(C)** of 23 plant roots. Al: *Ajuga lupulina*; Lr: *Lamiophlomis rotata*; Dh: *Dracocephalum heterophyllum*; Lt: *Lancea tibetica*; Pd: *Plantago depressa*; Sa: *Saussurea tibetica*; Tt: *Taraxacum tibetanum*; An: *Artemisia nanschanica*; Hs: *Heteropappus semiprostratus*; Lp: *Leontopodium pusillum*; Mk: *Morina kokonorica*; Ps: *Polygonum sibiricum*; St: *Stracheya tibetica*; Pb: *Potentilla bifurca*; Pa: *Potentilla anserina*; Pt: *Poa tibetica*; Sp: *Stipa purpurea*; En: *Elymus nutans*; Cm: *Carex moorcroftii*; Kt: *Kobresia tibetica*; Kp: *Kobresia pygmaea*; Kh: *Kobresia humilis*; Ip: *Iris potaninii*.

**FIGURE 3:**
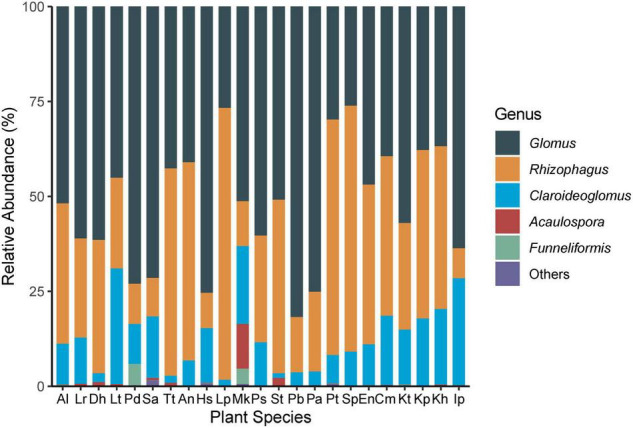
Composition of AMF genera in roots of 23 plant roots. Al: *Ajuga lupulina*; Lr: *Lamiophlomis rotata*; Dh: *Dracocephalum heterophyllum*; Lt: *Lancea tibetica*; Pd: *Plantago depressa*; Sa: *Saussurea tibetica*; Tt: *Taraxacum tibetanum*; An: *Artemisia nanschanica*; Hs: *Heteropappus semiprostratus*; Lp: *Leontopodium pusillum*; Mk: *Morina kokonorica*; Ps: *Polygonum sibiricum*; St: *Stracheya tibetica*; Pb: *Potentilla bifurca*; Pa: *Potentilla anserina*; Pt: *Poa tibetica*; Sp: *Stipa purpurea*; En: *Elymus nutans*; Cm: *Carex moorcroftii*; Kt: *Kobresia tibetica*; Kp: *Kobresia pygmaea*; Kh: *Kobresia humilis*; Ip: *Iris potaninii*.

**FIGURE 4:**
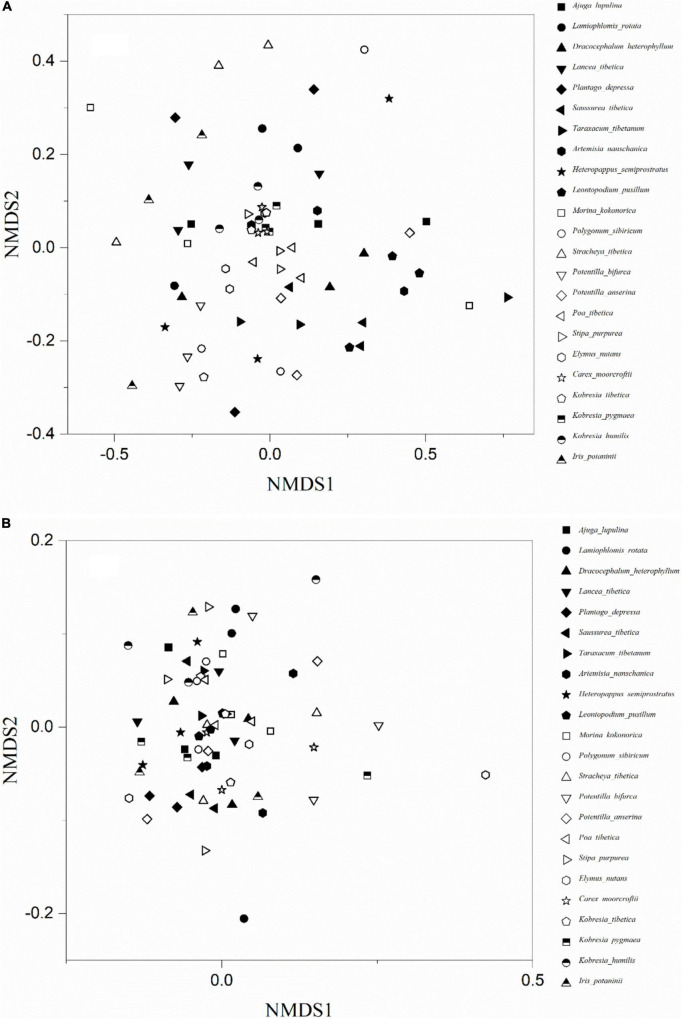
The non-metric multidimensional scaling (NMDS) ordinations of AMF taxonomic **(A)** and phylogenetic **(B)** community composition of 23 plant roots.

Arbuscular mycorrhizal fungi colonization was significantly different (*F* = 9.14, *P* < 0.001) among the 23 plant species (Figure 2A). The colonization of Hs was the lowest (18.3 ± 3.0%) and *Lancea tibetica* (Lt) was the highest (90.0 ± 2.7%). Significant differences in the Shannon-Wiener index were found among the AMF community hosted by different plant species (Figure 2B; *F* = 3.07, *P* = 0.001), but no significant differences were found in OTU richness (Figure 2C; *F* = 1.55, *P* = 0.10). *Elymus nutans* (En) of Poace had the highest AMF richness and Shannon-Wiener index, while *Leontopodium Pusillum* (Lp) of Asteraceae showed the lowest diversity. For example, *Glomus* and *Rhizophagus* were more enriched in *Potentilla bifurca* (Pb) and Lp root separately relative to other species, respectively (Figure 3). PERMANOVA showed that taxonomic composition was significantly different (Figure 4A; *F* = 2.81, *P* = 0.001), but not phylogenetic composition (Figure 4B; *F* = 1.38, *P* = 0.18) of AMF communities varied among plant species.

### Phylogenetic Correlations Between Plants and Arbuscular Mycorrhizal Fungi

**FIGURE 5:**
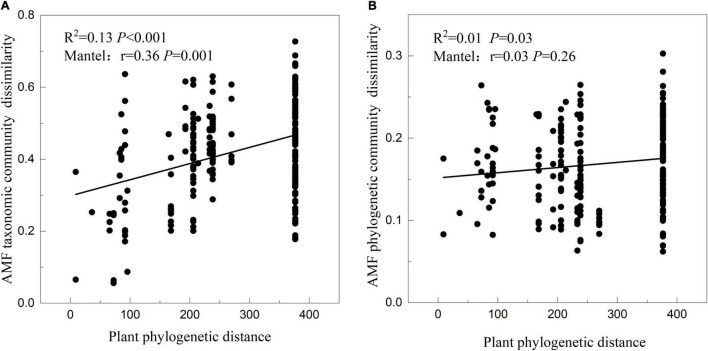
Relationships between the phylogenetic distance of 23 plant species and their corresponding AMF taxonomic **(A)** and phylogenetic **(B)** community dissimilarity tested by linear regression and mantel test.

The result of linear fitting and mantel test showed that there was a significant positive correlation between the plant interspecies phylogenetic distance and the corresponding AMF taxonomic community dissimilarity (Figure 5A; *R*^2^ = 0.13, *P* < 0.001; *r* = 0.36, *P* = 0.001), meaning that closely related plant species in phylogenetic trees tend to have more similar AMF community compositions. The result of linear fitting between the plant interspecies phylogenetic distance and corresponding AMF phylogenetic community dissimilarity was the same as above, but the mantel test was not significant (Figure 5B; *R*^2^ = 0.01, *P* = 0.03; *r* = 0.03, *P* = 0.26).

### Characteristics of Plant-AMF Network

**FIGURE 6:**
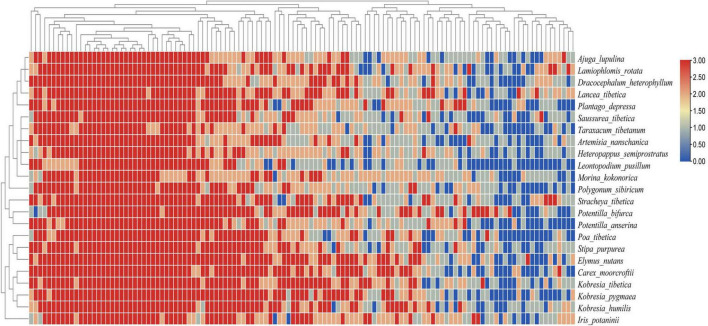
Heat map of plant-AMF association frequency matrix combined with the phylogenetic tree. Different shades of color denote the association frequency between different plant species and AMF OTUs, and blue denotes an absence of association.

**FIGURE 7:**
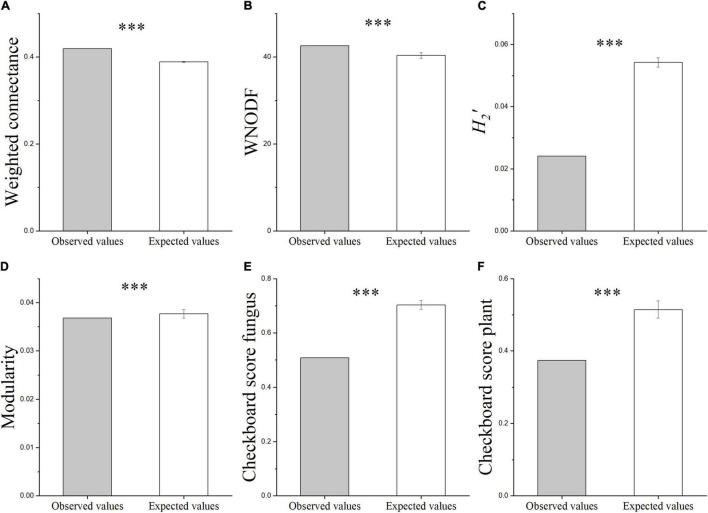
The characteristics of different plant-AMF networks. Weight connectance **(A)**, WNODF **(B)**, *H_2_′* **(C)**, modularity **(D)**, checkerboard score fungus [**(E)**, degree of avoiding overlapping of plants in the fungal community], and checkerboard score plant [**(F)**, degree of avoiding overlapping of fungi in the plant community]. Asterisks indicate significant differences between the observed and expected values according to the *t*-test (^***^P < 0.001; bars indicate SE).

The heat map of the plant-AMF incidence matrix showed that the symbiotic network relationship between the plant and AMF was established (Figure 6). Compared with the predicted value of the null model, the characteristics of the plant-AMF network, such as high connectance, high nestedness, anti-modularity, and anti-specialization, were obtained (Figures 7A–D). The observed values of weighted connectance and WNODF were significantly higher than the predicted values (*P* < 0.001). The values of *H_2_′* and modularity were both significantly lower than the observed values (*P* < 0.001). The results of the checkerboard score analysis showed that the observed values of plants and AMF communities were significantly lower than the expected values based on the null model (*P* < 0.001), but the checkerboard score (Figures 7E,F; fungus: 0.51, plant: 0.37) showed that the competition intensity of AMF was higher than that of plant species. The plant-AMF association frequency matrix showed that the phylogenetic signals of the plant species and AMF OTUs had significant effects on the plant-AMF network structure, and the phylogenetic signals from the plant were stronger than that from AMF (Table 1).

**TABLE 1:** Phylogenetic signals of plant-AMF based on incidence matrix frequency.

MSEd	MSEstar	MSEb	*d*plant (95% CI)	*d*AMF(95% CI)
0.26	0.32	0.46	0.41(0.36–0.45)	0.08(0.06–0.11)

*MSEd, mean-squared error (MSE) calculated for the full model; MSEstar, MSE calculated for a “star” phylogeny; MSEb, MSE calculated for a Brownian evolution model; d, the intensity of plant (dplant) and AMF (dAMF) phylogenetic signals.*

## Discussion

There were 121 AMF molecular species detected from plant roots in this study, indicating diverse AMF species in Tibetan alpine meadow, in concordance with the previous studies (Gai et al., 2009, 2012; Liu et al., 2011; Zhang et al., 2016). At the same time, it was consistent with previous studies (Gai et al., 2012; Li et al., 2014) that Cyperaceae plants which were considered unable or not easy to form mycorrhizal fungi also had high AMF colonization (Figure 2A; 40.4–60.2%) and diversity (Figures 2B,C; Shannon-Wiener: 2.31–2.60; richness: 86.67–90.67). These findings suggest the potential ecological importance of AMF for plant growth in the alpine meadow. To survive in the harsh environment of the alpine meadow, plants might choose AMF species to improve their ability to absorb nitrogen and phosphorus or the stress-resistance ability (Bever, 2002). It has been found that some AMF species with strong cold resistance ability could survive, thus affecting the growth of host plants and community dynamics (Deepika and Kothamasi, 2015; Bauer et al., 2017).

Furthermore, this study found a significant positive correlation between plant phylogenetic distance and AMF community dissimilarity. Thus, the selectivity existed in the plant-AMF interactions, so that closely phylogenetic-related plant species select similar AMF communities (Figure 5). This plant-AMF selectivity has been well documented in previous studies (Vandenkoornhuyse et al., 2003; Horn et al., 2014; Veresoglou and Rillig, 2014; Chagnon et al., 2015; Deepika and Kothamasi, 2015). The different AMF communities in the rhizosphere of different plant species may be achieved by preferentially allocating plant carbon to the most beneficial fungal partners (Bever et al., 2009; Kiers et al., 2011). The construction of the plant-AMF community was to maximize the functional matching between partners (Thompson, 2005). Plants with similar traits preferentially hosted similar AMF and, likewise, phylogenetically related AMF (assumed to have similar functional traits) interacted with similar plants. No doubt that the plant phylogeny was used to map the mycorrhizal information to examine evolutionary patterns (Wang and Qiu, 2006), but the complex symbiotic relationships between plants and AMF cannot be fully explained by plant phylogeny alone. The phylogenetic correlations between the interacting plant and AMF were modified by the competitive intransitivity (Laird and Schamp, 2006) and AMF species competition within a plant host (Roger et al., 2013). Therefore, future studies should involve factors, such as plant and AMF functional traits, species competition, climates, and soil nutrients, which can well explain the AMF community dynamics.

The network structure of 23 common plants and AMF was characterized by high connectance, high nestedness, anti-modularity, and anti-specialization, which was in line with the characteristics of the woody plant-AMF network in a subtropical forest in China (Chen et al., 2017). Generally, with the increase of connectance, the nestedness increased and the modularity decreased (Põlme et al., 2018), while the nestedness decreased, the modularity and the specialization increased (van der Heijden et al., 2015), as found in our network. High network connectance and nestedness and low network modularity indicated less segregation or sparse connections within subnetworks and dense connections between subnetworks for the plant-AMF network. These network properties enhanced the stability of the communities in the mutually beneficial network, which was considered to be an important property to promote the coexistence of species in the mutual system (Thebault and Fontaine, 2010). Theoretical studies suggested that the common nestedness patterns in the species network can determine the feasibility, resilience, durability, and structural stability of ecological communities (Toju et al., 2014), and the higher the nestedness of the interaction network, the better it can adapt to the effect of environmental disturbance.

In the analysis of plant-AMF phylogenetic signals based on the association frequency matrix, the results showed that the phylogenetic signals of plants and AMF had significant effects on the structure of the plant-AMF network. The phylogenetic signal from plants was stronger than that from AMF (Table 1). Close relatives of plants often interacted with AMF OTU species in the same group, but the interaction between AMF and plant species had a low correlation with AMF phylogeny, which was consistent with the study by Chen et al. (2017). In contrast, findings by Montesinos-Navarro et al. (2012a,b) showed that AMF phylogenetic diversity might also explain that there were different plant-AMF network structures in different ecosystems. In the AMF community with a more diverse phylogeny, there were more opportunities for specific AMF to coexist with common host plants, thus forming a highly nested symbiotic network structure (Chen et al., 2017). The phylogeny of plants and AMF played an important role in the non-random pattern of the AMF network in subtropical forest sites (Chen et al., 2017). In addition, the plant root traits in subtropical forests also showed strong interspecific phylogenetic signals (Kong et al., 2014). The modularity and nestedness in the plant-AMF network were caused by a variety of ecological processes, such as habitat heterogeneity (Olesen et al., 2007), specific selection of plant and AMF combination, relative species abundance and phylogenetic diversity, or AMF competition within the root (Verdu and Valiente-Banuet, 2011). The complex symbiotic interaction between plants and AMF has a potential effect on community assembly. Our study found that the characteristics of the plant-AMF network with high connectance and nestedness promote the stable symbiosis and interdependence between plants and AMF in Tibetan alpine meadows. Plants established network relationships with underground AMF through selection, which may affect the nutrient cycle of symbionts and even the succession of grassland community, so as to improve the adaptation to alpine ecological environment and stress. Therefore, further studies are required to elucidate the reciprocal symbiotic selection strategy, the coexistence pattern, community construction mechanism, and community dynamic rules of plant and AMF species. Furthermore, to enhance our understanding of the influence factors in the coevolution of plant-AMF is of great significance to study community structure, species evolution, and biodiversity.

## Conclusion

This study assesses the relationship between the common plant phylogeny and AMF and the characteristics of the plant-AMF symbiotic network in a Tibetan alpine meadow. There was a significant positive correlation between the phylogenetic distance of the 23 herbs and their AMF community dissimilarity, indicating the selectivity of plant species on their AMF community. The network structure of the plant and AMF was characterized by high connectance, high nestedness, anti-modularity, and anti-specialization, which potentially promoted the stable symbiosis and interdependence between plants and AMF in Tibetan alpine meadows. The plant-AMF network was affected by the plant-AMF phylogenies, and the phylogenetic signal from plants was stronger than that from AMF. This study emphasizes that plant phylogeny and plant-AMF network play an important role in the complex plant-AMF community mechanism and enhance our understanding of the interaction between aboveground and belowground communities.

## Data Availability Statement

The datasets presented in this study can be found in online repositories. The names of the repository/repositories and accession number(s) can be found below: https://www.ncbi.nlm.nih.gov/, PRJNA748864.

## Author Contributions

QD, BJ, and YL designed the experiments, which were then carried out by QD, KC, and SR. QD, XG, and YL collected the data and conducted the statistical analyses. QD wrote the first draft of the manuscript. QD, MM, JZ, YL, and BJ reviewed and edited the manuscript before submission. All authors made substantial contributions to the discussion of the content.

## Conflict of Interest

The authors declare that the research was conducted in the absence of any commercial or financial relationships that could be construed as a potential conflict of interest.

## Publisher’s Note

All claims expressed in this article are solely those of the authors and do not necessarily represent those of their affiliated organizations, or those of the publisher, the editors and the reviewers. Any product that may be evaluated in this article, or claim that may be made by its manufacturer, is not guaranteed or endorsed by the publisher.

## Footnotes

1
http://phylodiversity.Net/phylomatic/

2
http://maarjam.botany.ut.ee
