# Supplementary material for: Phylogenetic Correlation and Symbiotic Network Explain the Interdependence Between Plants and Arbuscular Mycorrhizal Fungi in a Tibetan Alpine Meadow
Source: Front Plant Sci. 2021 Dec 17;12:804861. doi: 10.3389/fpls.2021.804861 (PMC8718876; doi:10.3389/fpls.2021.804861)
Supplement: Supplementary file 1 [file Data_Sheet_1.docx]

**Figure S1 AMF OTUs sequencing rarefaction curve of different plant roots in Tibetan alpine meadow**

**
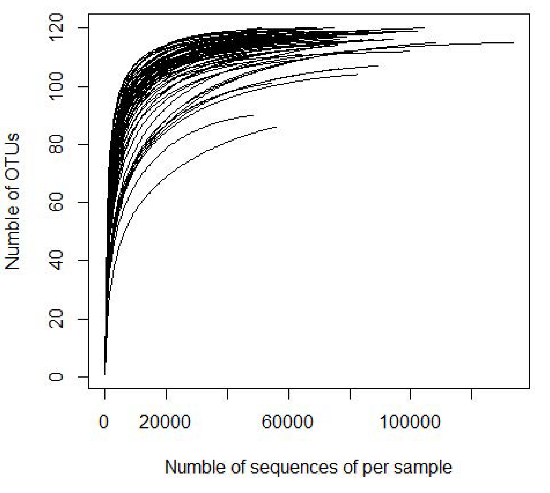
**

**
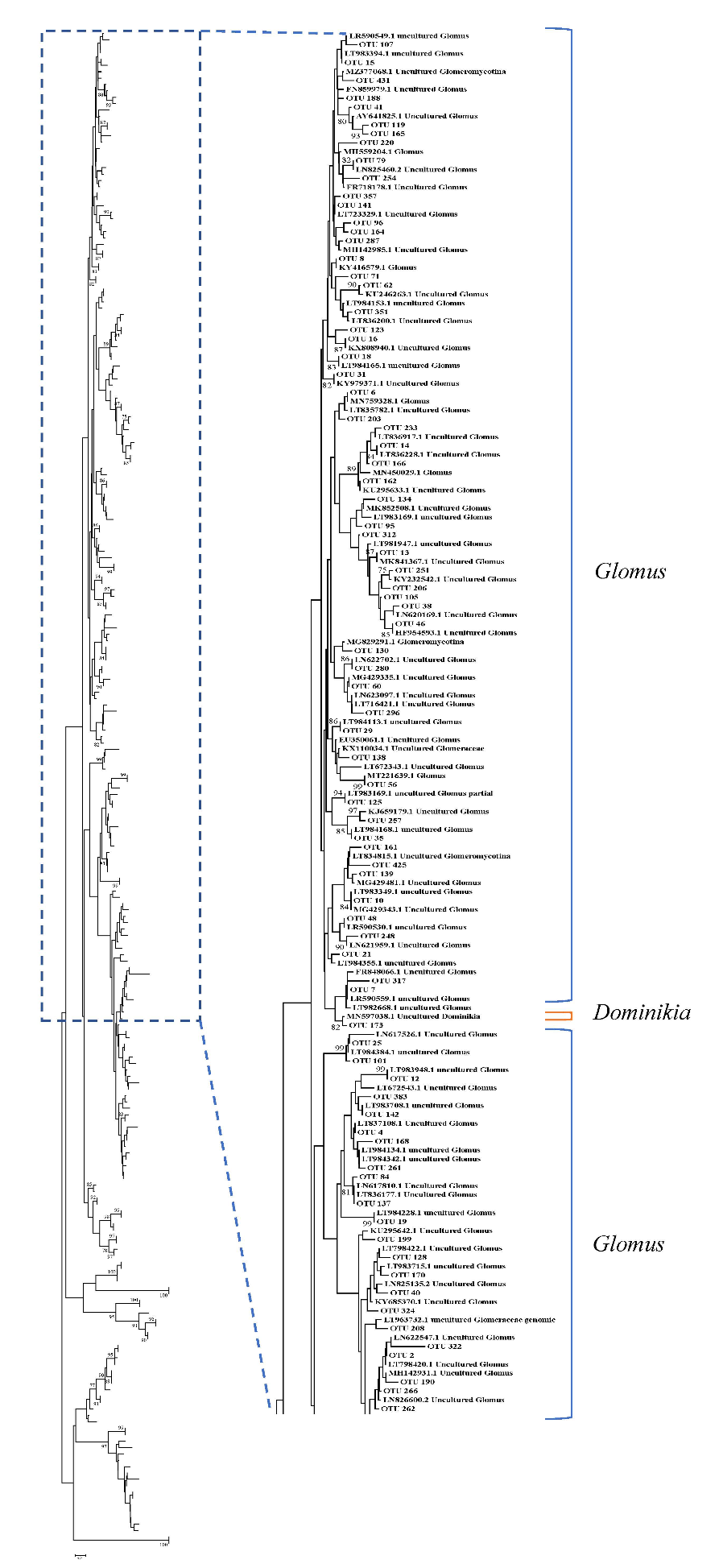
Figure S2 AMF OTUs phylogenetic tree of 23 plant roots in Tibetan alpine meadow**

**
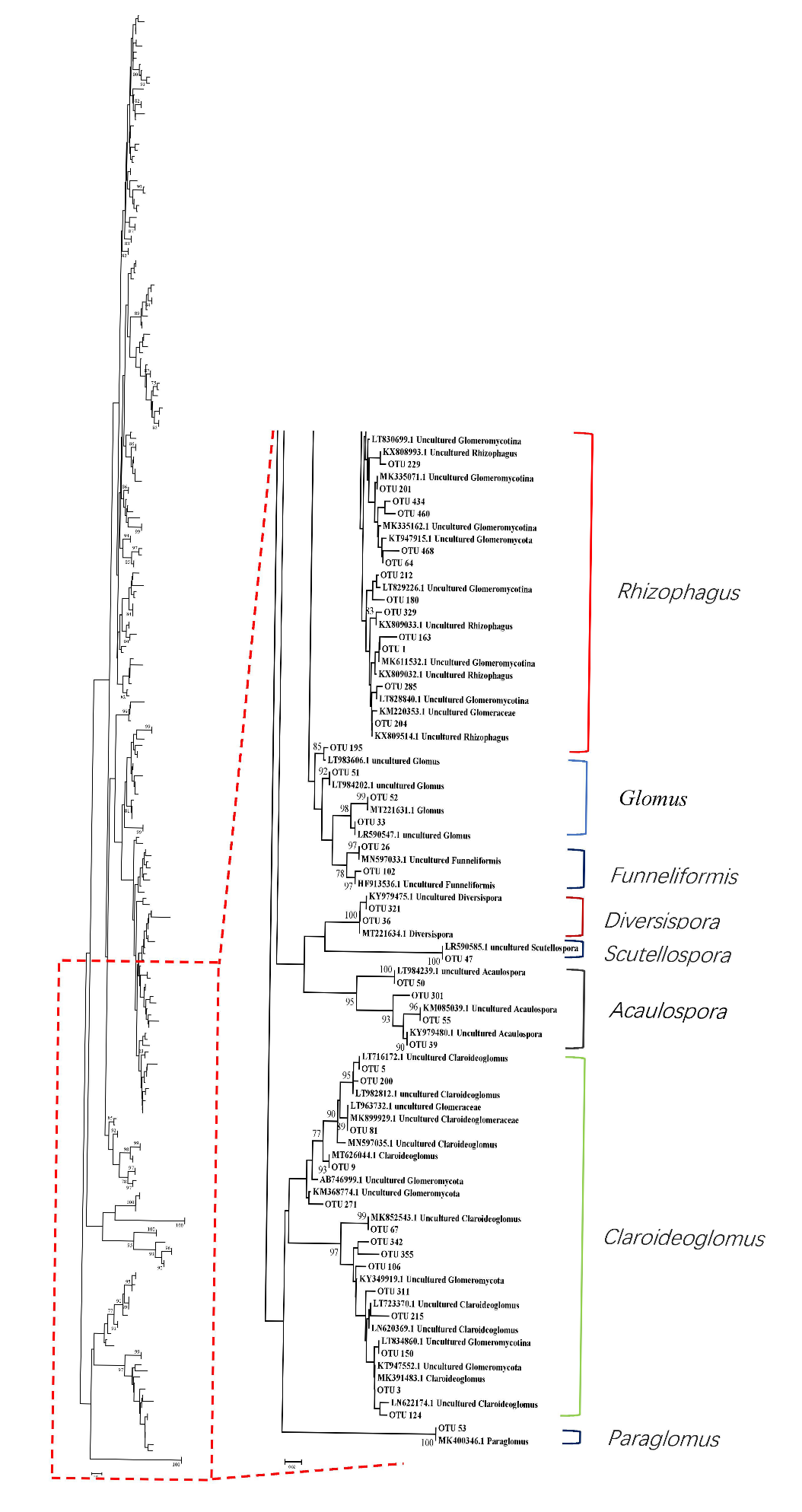
**



**Figure S3 AMF OTUs genera percentage composition of different plants in Tibetan alpine meadow**

**Table S1 AMF OTUs information of different plants in Tibetan alpine meadow**

| Order（3） | Families (6) | Genus (9) | OTU richness | Reads |
| --- | --- | --- | --- | --- |
| Glomerales | Claroideoglomeraceae | *Claroideoglomus* | 14 | 40 919 |
|  | Glomeraceae | *Dominikia* | 1 | 106 |
|  |  | *Funneliformis* | 2 | 1782 |
|  |  | *Glomus* | 84 | 181 013 |
|  |  | *Rhizophagus* | 12 | 117 836 |
| Diversisporales | Acaulosporaceae | *Acaulospora* | 4 | 2662 |
|  | Diversisporaceae | *Diversispora* | 2 | 474 |
|  | Gigasporaceace | *Scutellospora* | 1 | 80 |
| Paraglomerales | Paraglomeraceae | *Paraglomus* | 1 | 128 |
| Total reads | | | 121 | 345 000 |
