# Supplementary material for: Phylogenetic Correlation and Symbiotic Network Explain the Interdependence Between Plants and Arbuscular Mycorrhizal Fungi in a Tibetan Alpine Meadow
Source: Front Plant Sci. 2021 Dec 17;12:804861. doi: 10.3389/fpls.2021.804861 (PMC8718876; doi:10.3389/fpls.2021.804861)
Supplement: Supplementary file 2 [file Data_Sheet_2.docx]

**The ecological meaning of plant-AMF network index**

Connectance was defined as the ratio of the total number of actual network connections to the total number of possible network connections (Chen et al., 2017), where the species level matrix was calculated by weighted connectance. The value of weighted connectance ranged from 0 (no association among species) to 1 (association among all species). Network nestedness was measured by weighted nestedness metric based on overlap and diminishing fill (WNODF). The concept of nestedness described a special interaction pattern, in which the species with less interaction was a subset of the species with more interaction (Barberan et al., 2012). Nestedness mechanism could reduce interspecific competition and promote multi species coexistence, with high stability and durability (Bastolla et al., 2009). The value of WNODF ranged from 0 (completely random and disorder matrix) to 100 (fully nested structure). Modularity was a measure of the degree to which the network was divided into a small pattern of subset, in which the interaction density within a subset was higher than that between subsets (Olesen et al., 2007). The value of modularity was between 0 (random network without modularity) and 1 (maximum modularity). The specialization was indicated by *H_2_′* index, and was estimated based on the deviation between the actual number of interactions of a species and the expected total number of interactions of each species (Bluthgen et al., 2006). For a given total number of interactions, H2′ ranged from 0 (no specialization) to 1 (full specialization). Species co-occurrence pattern was used Checkboard score to indicate the randomness of the distribution of two or more species in the habitat. It was an effective method to infer the potential interaction (competition or mutual benefit) between organisms (Stone and Roberts, 1990). When the Checkboard score was very low, it meant that the distribution of species was of high randomness, that was, the distribution of one species was not affected by the distribution of other species, and high Checkboard score corresponded to high-intensity interspecific competition.

**REFERENCES**

Barberan, A., Bates, S.T., Casamayor, E.O., and Fierer, N. (2012). Using network analysis to explore co-occurrence patterns in soil microbial communities. *ISME J* 6(2), 343-351. doi: 10.1038/ismej.2011.119.

Bastolla, U., Fortuna, M.A., Pascual-Garcia, A., Ferrera, A., Luque, B., and Bascompte, J. (2009). The architecture of mutualistic networks minimizes competition and increases biodiversity. *Nature* 458(7241), 1018-1020. doi: 10.1038/nature07950.

Bluthgen, N., Menzel, F., and Bluthgen, N. (2006). Measuring specialization in species interaction networks. *BMC Ecol* 6, 1-12. doi: 10.1186/1472-6785-6-9.

Chen, L., Zheng, Y., Gao, C., Mi, X.C., Ma, K.P., Wubet, T., et al. (2017). Phylogenetic relatedness explains highly interconnected and nested symbiotic networks of woody plants and arbuscular mycorrhizal fungi in a Chinese subtropical forest. *Mol Ecol* 26(9), 2563-2575. doi: 10.1111/mec.14061.

Olesen, J.M., Bascompte, J., Dupont, Y.L., and Jordano, P. (2007). The modularity of pollination networks. *Proc. Natl. Acad. Sci. U.S.A.* 104(50), 19891-19896. doi: 10.1073/pnas.0706375104.

Stone, L., and Roberts, A. (1990). The checkerboard score and species distributions. *Oecologia* 85(1), 74-79. doi: 10.1007/bf00317345.
